# Prevalence of Symptomatic Established Rectus Diastasis of Parity in Primiparous Women: A Prospective Cohort Study From Early Pregnancy to 1‐Year Postpartum

**DOI:** 10.1002/wjs.70227

**Published:** 2026-01-08

**Authors:** Siobhan Elizabeth Fitzpatrick, Tamara Crittenden, David I. Watson, Nicola R. Dean

**Affiliations:** ^1^ College of Medicine and Public Health Flinders University Adelaide South Australia Australia; ^2^ Department of Plastic and Reconstructive Surgery Flinders Medical Centre Adelaide South Australia Australia; ^3^ Department of Surgery Flinders Medical Centre Adelaide South Australia Australia

**Keywords:** back pain, health‐related quality of life, postpartum, rectus diastasis

## Abstract

**Introduction:**

Rectus diastasis of parity is the separation of the abdominal muscles that can occur after childbearing. We hypothesized that a subpopulation of women with rectus diastasis also present with back pain and/or urinary incontinence, a condition referred to as symptomatic established rectus diastasis, and this results in impaired health‐related quality of life. This study identified the prevalence of symptomatic established rectus diastasis in primiparous women and measured their health‐related quality of life.

**Methods:**

Gravid nulliparous women over 18 years old were consecutively recruited from December 2021 to August 2022 and followed prospectively. Inter‐rectus distances were measured with ultrasound in early pregnancy, and 6‐week, 6‐month, and 12‐month postpartum. Patient reported outcome measures included the Oswestry Disability Index (ODI) for back pain, International Consultation on Incontinence Questionnaires Urinary Incontinence Short Form (ICIQ‐UI SF) for urinary incontinence, and the 36‐item short form (SF‐36) for health‐related quality of life.

**Results:**

Two‐hundred and thirteen women were recruited, of which 192 underwent ultrasound measurement in early pregnancy, 130 at 6‐week postpartum, 120 at 6‐month, and 109 at 12‐month. There was a significant increase in mean inter‐rectus distance over the study period (*p* < 0.001). The proportion of women with rectus diastasis at 12‐month postpartum (> 30 mm) was 30.3% and compared to those without they had worse back pain (*p* = 0.014) but no difference in urinary incontinence (*p* > 0.05). Women with symptomatic established rectus diastasis at 12‐month postpartum (rectus diastasis and back pain (ODI > 0)), made up 25% of the cohort and had significantly worse health‐related quality of life than those without (*p* < 0.05). Predictive factors for symptomatic established rectus diastasis included increased total fetal birthweight (OR 3), lower maternal BMI (OR 1.2), and gestational diabetes (OR 6.7).

**Conclusion:**

This study of gravid nulliparous women from early pregnancy until 12‐month postpartum identified rectus diastasis in 30.3% and symptomatic established rectus diastasis in 25%. Women with symptomatic established rectus diastasis had significantly worse health‐related quality of life.

## 1 Introduction

Rectus diastasis of parity is the separation of the rectus abdominis muscles that occurs in relation to childbearing. During pregnancy, the anterior abdominal wall, comprised of its thickened midline of connective tissue, the linea alba, and its associated muscular attachments, elongates and widens under the influence of hormones and mechanical stress to accommodate the growing fetus [1]. After birth, the abdominal wall usually undergoes contraction to return to close to its prepregnancy state within the first postpartum year. However, in a proportion of women, this does not occur and the linea alba and abdominal wall remains stretched. The prevalence of established rectus diastasis of parity at 1‐year postpartum has been estimated at approximately one in three women [2]. What is yet to be elucidated is the clinical relevance of this state. There is growing evidence that rectus diastasis of parity is associated with symptoms such as back pain [3], urinary incontinence [4], abdominal dysfunction [5, 6], and impaired health‐related quality of life [7]. This has significant implications for women's health. We hypothesized that there is a specific subpopulation of women with measurable rectus diastasis that also present with symptoms, such as back pain and urinary incontinence, and that these women will have worse health‐related quality of life compared to those without.

This aim of this study was to determine the prevalence of symptomatic rectus diastasis in primiparous women and to determine their health‐related quality of life compared to women without rectus diastasis.

## 2 Methods

### 2.1 Recruitment and Timeline

Ethical approval was provided by the Southern Adelaide Clinical Human Research Ethics Committee (Approval number 162.21). Women over 18 years old were recruited consecutively from the Southern Adelaide Local Health Network antenatal clinic from December 2021 to August 2022. Participants were gravid nulliparous women who were less than 16‐week gestation who consented to participate. Women with connective tissue disorders (e.g., Ehler's Danlos Syndrome), previous major abdominal surgery, or had progressed beyond 16‐week gestation were excluded. Women with significant pregnancy complications, premature delivery (32‐week), or miscarriage after recruitment were excluded from analysis. Women were followed up at 6‐week postpartum, 6‐month postpartum, and 12‐month postpartum.

### 2.2 Questionnaires and Medical Records Data Collection

At enrollment women completed an online demographic questionnaire including potential confounders for health‐related quality of life, such as smoking status, participation in paid work, or education level. Women were asked about engagement with physiotherapy or core exercise programs and breastfeeding status at each timepoint after birth. Delivery details were collected from electronic medical records after women confirmed their child's date of birth.

The patient reported outcome measures (PROMs) applied in this study were the Oswestry Disability Index (ODI) to assess back pain [8], the International Consultation on Incontinence Questionnaires Urinary Incontinence Short Form (ICIQ‐UI SF) to assess urinary incontinence [9], and the 36‐item Short Form Questionnaire (SF‐36) to assess health‐related quality of life [10]. As with the demographic questionnaire, these instruments were administered online via Research Electronic Data capture (REDCap) software (Vanderbilt University, T.N.; https://projectredcap.org). The PROMs were administered at baseline and each follow‐up timepoint.

### 2.3 Participant Measurement

Height and weight were measured at baseline. Inter‐rectus distances were measured using a General Electric Logiq V2 ultrasound machine with high resolution 40 mm linear array transducer (GE Healthcare Technologies Inc., Chicago, I.L.) using a predetermined protocol (Supporting Information S1). The threshold criteria for confirmation of rectus diastasis in this study was 30 mm. Women underwent inter‐rectus measurements at baseline and each follow‐up timepoint.

### 2.4 Statistical Analysis

An a priori power calculation was performed using PASS 2021 Power Analysis and Sample Size software (NCSS LLC. Kaysville, Utah) to determine the sample size required to detect differences in health‐related quality of life for women with versus without rectus diastasis at 12‐month. Parameters were applied for an analysis of covariance comparing two groups with and without diastasis. The estimated ratio of one to two women was derived from Sperstad et al. [2] and the difference in mean SF‐36 physical function scores of 92 and 85 was derived from Gitta et al. [7] After applying a power of 80%, *R* ^2^ of 0.1, significance of 0.05 and an attrition rate of 40% [2], the target sample size was determined to be 214.

Statistical analysis was conducted using IBM SPSS v29.0 statistical software (IBM Corp., Armonk, N.Y.). Descriptive statistics were presented as means and 95% confidence intervals (95% CIs) and frequency and percentages. Datasets were compared using independent *t*‐tests, chi‐squared, or Fisher's exact tests as appropriate. Outcome variables are reported as means and standard deviations (SDs). Distribution‐based minimum clinically important difference (MCID) of SF‐36 scores was set as half a pooled standard deviation (0.5 SD) [11]. Linear mixed models using participants as random effects, and time as a fixed effect, were used to estimate mean inter‐rectus distances. Multivariate linear regression and multiple logistic regression analyses were applied to determine significant predictors of inter‐rectus distance and symptomatic rectus diastasis, respectively. The logistic regression model followed an augmented backward elimination method of selection from significantly associated variables [12]. Significance was set at *p* < 0.05.

## 3 Results

### 3.1 Study Population

**FIGURE 1:**
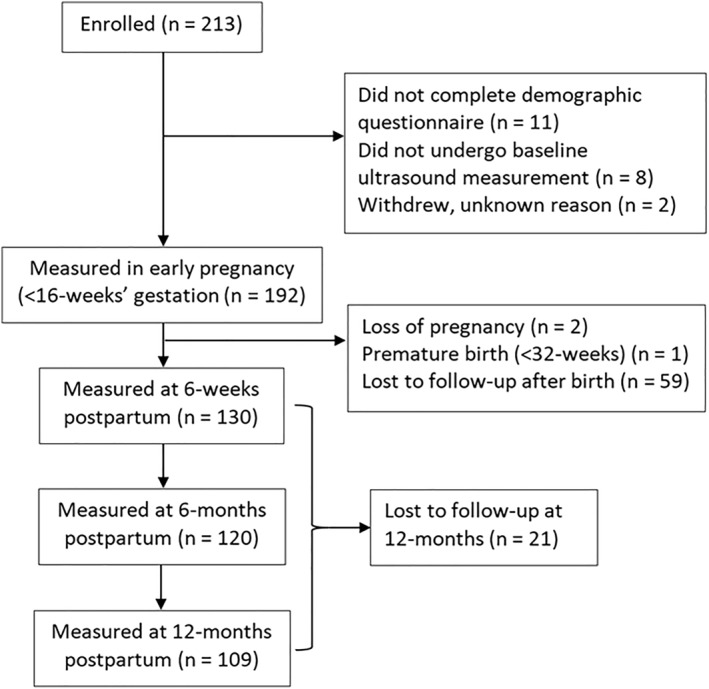
Flow of participants from enrollment to final follow‐up at 12‐month postpartum.

Two‐hundred and thirteen nulliparous women were enrolled before 16 weeks of pregnancy, of which 192 underwent ultrasound measurement in early pregnancy. Figure 1 summarizes the participant numbers at various timepoints.

Population characteristics are summarized in Table 1. The sample was representative of the local, state, and national population norms for women giving birth for age, BMI, country of birth, and use of assisted reproductive technology and twin pregnancies [13]. However, the number of Aboriginal or Torres Strait Islander women was significantly lower (Table S1).

**TABLE 1:** Participant characteristics of sample population (*n* = 189).

Characteristic	Frequency (%)
Biometric variables	
Age at delivery—mean (SD; range) (years)	30.5 (4.8; 20–43)
Height—mean (SD; range) (cm)	164.8 (6.5; 150–185)
Weight—mean (SD; range) (kg)	71.6 (16.2; 44.5–142)
BMI—mean (SD; range) (kg/m^2^)	26.3 (5.7; 18–59.9)
Demographic variables	
Aboriginal or Torres Strait Islander status	
Nonaboriginal or Torres Strait Islander	179 (94.7)
Aboriginal or Torres Strait Islander	1 (0.5)
Not stated	9 (4.8)
Country of birth	
Australia	133 (70.7)
Other	55 (29.3)
Missing	1 (0.5)
Ethnicity	
Caucasian	152 (80.9)
Non‐Caucasian	36 (19.1)
Missing	1 (0.5)
Language spoken at home	
English	171 (91)
Other	17 (9)
Missing	1 (0.5)
Education level	
Secondary	40 (21.6)
Tertiary	145 (78.4)
Missing	4 (2.1)
Involved in paid work	
No	10 (5.5)
Yes	173 (94.5)
Missing	6 (3.2)
Health variables	
Smoking status	
Never	164 (87.7)
Previously	21 (11.2)
Yes	2 (1.1)
Missing	2 (1.1)
Reproductive variables	
Gestational diabetes	
Yes	27 (14.3)
No	162 (85.7)
Assisted reproductive technology	
Yes	11 (5.8)
No	178 (94.2)
Multiples	
Singleton	185 (97.9)
Twins	4 (2.1)

Women were more likely to be lost to follow‐up if they were non‐Caucasian ethnicity (*p* = 0.01), non‐English speaking at home (*p* = 0.047), previous or current smokers (*p* = 0.047), and if they did not participate in paid work (*p* = 0.017). Whether a woman developed rectus diastasis did not influence likelihood of follow‐up (*p* > 0.05) (Table S2).

### 3.2 Delivery Characteristics

Delivery characteristics are summarized in Table S3. The rates of birth by caesarean section, spontaneous vaginal, and instrument‐assisted were comparable to the national rates [14] (Table S4). Rates of induction were slightly higher than the state and national averages, and rates of third‐ and fourth‐degree perineal tears were slightly lower.

### 3.3 Anatomical Site and Prevalence of Measured Inter‐Rectus Diastasis

**FIGURE 2:**
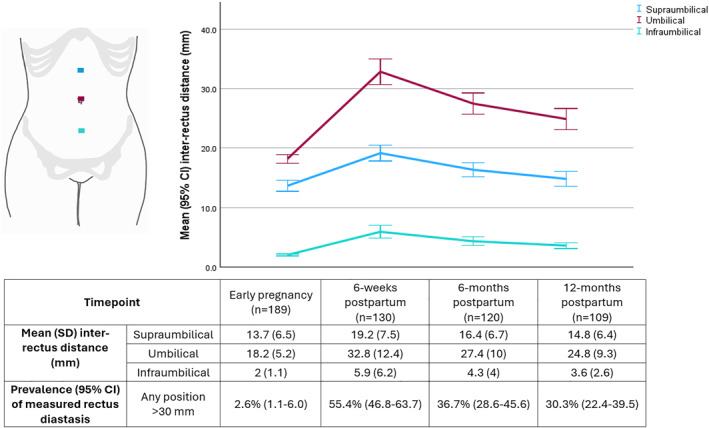
Inter‐rectus distances (mm) and prevalence (%) of measured rectus diastasis over time from early pregnancy until 12‐month postpartum.

The mean inter‐rectus distance for the three positions on the linea alba are summarized in Figure 2. At all three positions, mean inter‐rectus distance at all timepoints postpartum was significantly greater than at baseline in early pregnancy. There was a significant reduction in mean inter‐rectus distance from 6‐week postpartum to 6‐month, but not from 6‐month to 12‐month postpartum, indicating that the reduction in diastasis did not continue beyond 6 months. The widest inter‐rectus distances were consistently found to be at the umbilical level on measurement. The proportion of women who exhibited rectus diastasis (> 30 mm) is shown in Figure 2, with 99% of cases surpassing this threshold at the umbilical level. The cases measured in early pregnancy were in either twin pregnancies with early abdominal wall expansion or a case of preexisting supraumbilical rectus diastasis also present at the level of the costal margin, described by Nahas as a congenital variation [15].

### 3.4 Severity and Prevalence of Back Pain and Urinary Incontinence

Across the whole cohort, women reported significantly more back pain a year postpartum compared to baseline during early pregnancy, although for the whole group the effect size was trivial (Cohen's *d* = 0.185 and 95% CI 0–0.369). Comparing those with measured rectus diastasis to those without, the women with rectus diastasis had a significant increase in back pain throughout the study period (*p* = 0.018, Cohen's *d* = 0.393, and 95% CI 0.025–0.756), whereas those without rectus diastasis demonstrated no change.

When the back pain scores of women with measured rectus diastasis and no rectus diastasis were directly compared at 12‐month postpartum with an independent *t*‐test, the women with rectus diastasis had significantly higher back pain scores (*p* = 0.011) (Table 2). This was associated with a moderate Cohen's *d* effect size of −0.592 (95% CI −1.014 to −0.167).

**TABLE 2:** Back pain and urinary incontinence scores from early pregnancy until 12‐month postpartum and comparison of women with and without rectus diastasis at 12‐month postpartum on independent *t*‐test.

	Time point	ODI score				ICIQ‐UI SF score			
		*n*	Mean	SD	*p*	*n*	Mean	SD	*p*
Whole cohort	Early pregnancy	184	5.7	7.1		181	1.5	2.9	
	6‐week postpartum	134	9.4	9.7		133	2.5	3.6	
	6‐month postpartum	120	7.4	7.8		120	2.6	40	
	12‐month postpartum	117	6.9	7.7		116	2.7	3.5	
No diastasis vs. diastasis	12‐month postpartum	73 vs. 32	5.0 vs. 9.2	6.0 vs. 9.3	0.01	73 vs. 32	2.7 vs. 2.3	3.8 vs. 3.2	0.302

Urinary incontinence was more of a problem after giving birth than at baseline, with a significant difference in urinary incontinence scores between baseline and 12‐month postpartum (see Table 2). When urinary incontinence scores of women with and without measured rectus diastasis at 12‐month were compared using an independent *t‐*test, no significant difference was demonstrated (*p* = 0.302) (Table 2).

### 3.5 Prevalence of Symptomatic Established Rectus Diastasis and Health‐Related Quality of Life of Women

**FIGURE 3:**
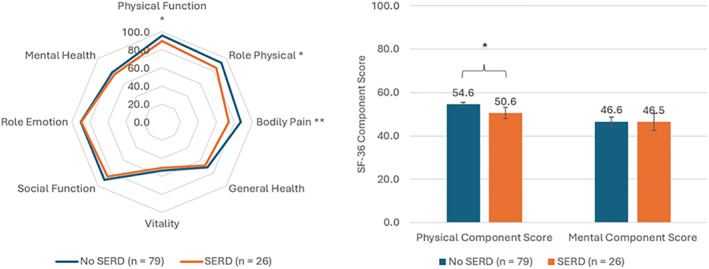
SF‐36 domain and component scores for women with and without symptomatic established rectus diastasis (SERD) at 12‐month postpartum.

Women who had measured rectus diastasis (> 30 mm) persisting at 12‐month and who also reported back pain (ODI score > 0), were considered to have symptomatic established rectus diastasis. As urinary incontinence scores were not significantly associated with measured rectus diastasis, they were not included in the criteria for symptomatic established rectus diastasis. Twenty‐six women in the population met these criteria at 12‐month postpartum, making up 24.7% of the cohort. The SF‐36 scores of women with and without symptomatic established rectus diastasis were compared with independent *t*‐tests (Figure 3 and Table S5). Women with symptomatic established rectus diastasis demonstrated worse health‐related quality of life, with significantly lower scores for physical function, role physical, bodily pain, and physical component score (*p* < 0.05). These differences also exceeded the 0.5 SD threshold for MCID, which is considered clinically meaningful to participants [11].

### 3.6 Risk Factors for Increasing Inter‐Rectus Distance and Symptomatic Established Rectus Diastasis

Gestation at delivery, failure to progress, and presence of a first‐ or second‐degree perineal tear significantly predicted umbilical inter‐rectus distance as a continuous measure at 12‐month, *F* (3, 104) = 8.03, *p* < 0.001, and *R* ^2^ of 0.165, explaining 16.5% of the variance in the data. Regression coefficients and standard errors are included in Table S6. For every additional week of gestation, the predicted inter‐rectus distance increased by 2 mm; if there was failure to progress during childbirth, the estimated distance increased by 5 mm; and if a first‐ or second‐degree perineal tear occurred, the estimated distance decreased by 5 mm.

On binomial logistics regression, gestational diabetes, total fetal birthweight, and maternal BMI significantly contributed to the model, which could successfully predict cases of symptomatic established rectus diastasis at 12‐month postpartum (*χ* ^2^ = 20.6 and *p* < 0.001) (Table S7). Women with gestational diabetes had 6.7‐times higher odds of developing symptomatic established rectus diastasis than those without (OR 6.66 and 95% CI 1.38–32.1). The heavier the baby at birth, the increased likelihood of symptomatic established rectus diastasis, with each additional kilogram increasing the likelihood by a factor of three (OR 3.06 and 95% CI 1.02–9.16). Conversely, lower maternal BMI (at baseline) was associated with an increased risk of the outcome (OR 1.2 and 95% CI 1.03–1.35). In the multivariate analysis, delivery method and failure to progress did not significantly contribute to the predictive model, indicating that caesarean section was not an independent risk factor for symptomatic established rectus diastasis in this study.

## 4 Discussion

This prospective longitudinal cohort study followed gravid nulliparous women from early pregnancy until 12‐month postpartum to observe changes in inter‐rectus distance. The study determined the proportion of women who developed rectus diastasis, denoted by a measured inter‐rectus distance of greater than 30 mm, identified risk factors for its development, and determined if a proportion of women developed concurrent symptoms, which we elected to refer to as symptomatic established rectus diastasis.

This is the first study to characterize the natural history of rectus diastasis in primiparous women with the gold‐standard methodology of ultrasound, in the same cohort of women before and after their first full‐term pregnancy, with satisfactory follow‐up, and validated outcome measures. This study contributes meaningfully toward the medical definition of rectus diastasis as a common condition that affects childbearing women globally, and the significant risk factors identified here could inform clinical practice.

Just over half of women had detectable rectus diastasis at the earliest check after delivery with this reducing to just under a third at a year post delivery. This is similar to the findings of Sperstad and Mota, although those studies had methodological limitations [2, 16]. The threshold for rectus diastasis employed in our study was 30 mm, which is a distance supported by other research [17].

In the present prospective study, women who had measured rectus diastasis of 30 mm or more at 12‐month postpartum had significantly worse back pain than women without rectus diastasis. The relationship between back pain and rectus diastasis in this cohort study is supported by findings in large cross‐sectional studies [3, 7]. The anatomical basis for this relationship has also been explored in biomechanical studies examining the effect of abdominal muscle function on spinal ligaments and intra‐discal pressures, and surgical studies examining the effects of abdominoplasty on spinal curvature [18, 19, 20, 21].

When parous women were compared at 12‐month postpartum based on the presence of measured rectus diastasis and back pain, there were statistically and clinically meaningful differences in their resultant health‐related quality of life scores. These women were deemed to have symptomatic established rectus diastasis, and they comprised approximately 25% of the population. From studies of the effects of abdominoplasty with repair of rectus diastasis, it is anticipated that these women might benefit from and be considered for surgical intervention, particularly if they have exhausted conservative therapeutic options [22, 23].

It is important to recognize the difference between broader phenomenon of measured rectus diastasis and symptomatic established rectus diastasis. Our study suggests that the natural reduction in inter‐rectus distance is unlikely to improve beyond 12‐month postpartum, and the proportion of women who developed this anatomical issue is in line with other studies in the literature. What has not been adequately characterized is the relationship that this anatomical condition has with symptoms such as back pain and health‐related quality of life. Even though the proportion of these women at 12‐month postpartum who were symptomatic was approximately one in four, the timeline for the progression of the symptoms may not end at 12‐month postpartum, and follow‐up beyond 12‐month was not part of this study. Although significant differences in back pain and health‐related quality of life were detected in our primiparous cohort, there is evidence that more severe symptoms can occur in multiparous women and at much later postpartum follow‐up than the duration of our study, and at least some of these individuals appear to benefit from surgical abdominal wall repair [22, 24]. Our cohort might reflect a milder or earlier stage of the clinical picture of women with symptomatic established rectus diastasis. To connect our findings with the more severe symptomatology reported in some multiparous women followed longer term, future high quality long‐term follow‐up studies of women who have completed their families are needed, even though they would be logistically challenging.

Although, it was anticipated that urinary incontinence would form part of the symptom profile of symptomatic established rectus iastasis, the present study failed to demonstrate a significant relationship and so it was not included in the clinical criteria. Other studies have also failed to demonstrate a clear relationship between urinary incontinence and rectus diastasis [17, 25, 26, 27]. Considering that nearly one third of women reported some urinary incontinence in early pregnancy, the causes of urinary incontinence are likely related to other issues during both pregnancy and within the first postpartum year [28, 29].

The finding in this study that long gestation, gestational diabetes, and failure to progress were significant predictive factors for symptomatic established rectus diastasis aligns with other studies [30, 31]. There are various hypothesized pathophysiological mechanisms by which gestational diabetes may influence rectus abdominis muscle, but its clinical impact on rectus muscle function and symptomatic established rectus diastasis requires further exploration [32, 33, 34, 35, 36]. In contrast to other studies, once the delivery modality was incorporated into the model with these other factors, delivery by caesarean section was not independently associated with an increased likelihood of symptomatic established rectus diastasis compared to vaginal delivery [31, 37].

The strengths of our study include the use of ultrasound to measure inter‐rectus distance, the use of validated PROMs to measure outcomes of interest, the application of an a priori power calculation to determine differences in health‐related quality of life at the study end point, assessment of potential confounders, and a length of follow‐up of 12‐month postpartum. This study is unique in examining the same gravid nulliparous women prospectively from early pregnancy before abdominal wall stretching and following the cohort recurrently for 12‐month postpartum. A potential limitation was the attrition rate from the study overall, although it was similar to comparable studies [2, 16] and participant retention in the postpartum period counterbalanced this. The generalizability of the results is limited to nulliparous women in a single Australian center, and by the higher attrition rate for women who are not involved in paid work, smokers, non‐Caucasian, and non‐English speaking at home.

Determination of the impacts of further postpartum time and additional childbearing in multiparous women would be a valuable contribution to future research. Multiparity has been demonstrated to increase the risk of rectus diastasis [2, 3, 7, 38, 39], and its impact on symptomatic established rectus diastasis will require a longer timeframe than applied in the current study.

## 5 Conclusion

This is the first study evaluating rectus diastasis in a large prospective population of gravid nulliparous women from early pregnancy to 12‐month postpartum. The prevalence of measured rectus diastasis at 12‐month postpartum was 30.3%. Twenty‐five percent of women were symptomatic and should be the focus for any future interventions. These results support considering the use of “Symptomatic Established Rectus Diastasis” to describe a discreet clinical entity, which can be defined as measured rectus diastasis with back pain. The women who met these criteria had statistically significant and clinically meaningful impairments in health‐related quality of life outcomes compared to the rest of the population. Significant risk factors for symptomatic established rectus diastasis were higher total fetal birthweight, lower maternal BMI, and gestational diabetes. Identification of women with these factors may enable focused intervention for rectus diastasis in the early postpartum period, and if conservative measures are unsuccessful surgical repair might be considered.

## Author Contributions

**Siobhan Elizabeth Fitzpatrick:** conceptualization (equal), investigation (lead), methodology (lead), project administration (lead), data curation (lead), formal analysis (lead), writing – original draft preparation, writing – review and editing (equal). **Tamara Crittenden:** supervision (equal), conceptualization (equal), validation (equal), writing – review and editing (equal). **David I. Watson:** supervision (equal), funding acquisition (equal), conceptualization (equal), validation (equal), writing – review and editing (equal). **Nicola R. Dean:** supervision (equal), conceptualization (equal), funding acquisition (equal), project administration (supporting), methodology (supporting), validation (equal), writing – review and editing (equal).

## Funding

The authors have nothing to report.

## Ethics Statement

Ethical approval was obtained by the Southern Adelaide Clinical Human Research Ethics Committee (Approval number 162.21).

## Consent

Informed consent was obtained from all individual participants included in the study.

## Conflicts of Interest

The authors declare no conflicts of interest.

## Supporting information


Supporting Information S1

**Table S1**: Comparison of participant characteristics with mean values in the population of birthing mothers in the local hospital network population, South Australian population, and Australian population [1].

**Table S2**: Likelihood of loss to follow‐up at 12‐month, stratified by baseline variables and inter‐rectus distance measurements at 6‐week and 6‐month postpartum.

**Table S3**: Summary of delivery characteristics.

**Table S4**: Comparison of delivery characteristics of the sample population with state and national rates derived from the National Core Maternity Indicators report [1].

**Table S5**: SF‐36 scores for women with and without symptomatic established rectus diastasis at 12‐month postpartum.

**Table S6**: Multiple linear regression model for umbilical inter‐rectus distance at 12‐month postpartum.

**Table S7**: Binomial logistic regression of risk factors for symptomatic established rectus diastasis at 12‐month postpartum.

## Data Availability

The data that support the findings of this study are available from the corresponding author upon request. The data are not publicly available due to privacy or ethical restrictions.
