# Supplementary material for: Prevalence of Symptomatic Established Rectus Diastasis of Parity in Primiparous Women: A Prospective Cohort Study From Early Pregnancy to 1‐Year Postpartum
Source: World J Surg. 2026 Jan 8;50(2):344–52. doi: 10.1002/wjs.70227 (PMC12904848; doi:10.1002/wjs.70227)
Supplement: Supplementary file 1 — Supporting Information S1 [file WJS-50-344-s001.docx]

Supplementary File 1. Inter-rectus distance measurement protocol

Inter-rectus distance was measured using a General Electric Logiq® V2 ultrasound machine with high resolution 40 mm linear array transducer (GE Healthcare Technologies Inc., Chicago, I.L.). Measurements were taken by the researcher (SF) whilst participants were supine, relaxed, and at end expiration. Inter-rectus measurements were recorded at three points along the linea alba: (1) halfway from xiphisternum to umbilicus; (2) at the superior border of the umbilicus; and (3) halfway from the umbilicus to the pubic symphysis. The ultrasound probe was used transversely and held perpendicular to the skin with minimal downward pressure to avoid distortion of the fascial layers beneath. Images were obtained in B-mode. After static images were obtained at the three points described above, on-screen callipers were applied to measure the distance from the medial aspect of each rectus abdominis muscle.

To accommodate the greater inter-rectus distances associated with pregnancy and the postpartum period, either an “extended field-of-view” mode or an acoustic standoff pad were utilised for ultrasound measurement, both of which have previously been demonstrated to have excellent correlation with standard views (1). If the medial edges of the rectus muscles could not be viewed in a single frame using either of these methods, usually for widths of over 40mm, a metal frame was used to provide a midline surface marker, and side by side images were taken. When the callipers were applied to each of these images, the lateral marker was placed at the medial edge of the muscle, and the medial marker was placed at the same depth but perpendicular to the metal surface marker. Depth and gain were adjusted as required to optimise all images.

References

1. Keshwani N, Mathur S, McLean L. Validity of Inter-rectus Distance Measurement in Postpartum Women Using Extended Field-of-View Ultrasound Imaging Techniques. J Orthop Sports Phys Ther. 2015;45(10):808-13. Epub 20150824. doi: 10.2519/jospt.2015.6143. PubMed PMID: 26304645.
