# Supplementary material for: Prevalence of Symptomatic Established Rectus Diastasis of Parity in Primiparous Women: A Prospective Cohort Study From Early Pregnancy to 1‐Year Postpartum
Source: World J Surg. 2026 Jan 8;50(2):344–52. doi: 10.1002/wjs.70227 (PMC12904848; doi:10.1002/wjs.70227)
Supplement: Supplementary file 2 — Table S1: Comparison of participant characteristics with mean values in the population of birthing mothers in the local hospital network population, South Australian population, and Australian population [1]. [file WJS-50-344-s007.docx]

Supplementary Table 1. Comparison of participant characteristics with mean values in the population of birthing mothers in the local hospital network population, South Australian population, and Australian population (1)

| Characteristic | Sample^ | SALHN^ | SA | Australia |
| --- | --- | --- | --- | --- |
| *N* | 189 | 1,748 | 8,526^ | 135,205^ |
| Mean age (years) | 30.5 | 29.7* | NA | 29.7^* |
| Mean BMI (kg/m^2^) | 26.3 | 27.1 | 27.2* | 26.4 |
| BMI categories |  |  |  |  |
| Underweight (<18.5) | 1.1% | 1.8% | 1.8% | 3.0% |
| Normal weight (18.5-24.9) | 48.1% | 40.8% | 41.6% | 46.6% |
| Overweight (25-29.9) | 31.6% | 32.0% | 29.7% | 27.7% |
| Obese (>30) | 19.3% | 25.3% | 26.9% | 22.7% |
| Country of birth (Australia) | 70.4% | NA | 72.7% | 65.6% |
| Aboriginal or Torres Strait Islander | 0.5% | 3.4%* | 4.20%* | 5.0%* |
| Non-Aboriginal or Torres Strait Islander | 99.5% | 96.6% | 95.8% | 95% |
| Assisted reproductive technology | 5.8% | NA | NA | 5.4% |
| Multiples | 1.9% | 1.7% | 1.4% | 1.4% |

^* One sample T-test or Chi-Square test,^ *^p^* ^<0.05
^ Population values are first-time mothers^

^n, Population number; BMI, Body Mass Index; SALHN, Southern Adelaide Local Health Network; SA, South Australia^

References

1. Australian Institute of Health and Welfare. Australia's mothers and babies. Canberra: AIHW, 2023.
