# Supplementary material for: Prevalence of Symptomatic Established Rectus Diastasis of Parity in Primiparous Women: A Prospective Cohort Study From Early Pregnancy to 1‐Year Postpartum
Source: World J Surg. 2026 Jan 8;50(2):344–52. doi: 10.1002/wjs.70227 (PMC12904848; doi:10.1002/wjs.70227)
Supplement: Supplementary file 3 — Table S2: Likelihood of loss to follow‐up at 12‐month, stratified by baseline variables and inter‐rectus distance measurements at 6‐week and 6‐month postpartum. [file WJS-50-344-s004.docx]

Supplementary Table 2. Likelihood of loss to follow-up at 12-months, stratified by baseline variables and inter-rectus distance measurements at 6-weeks and 6-months postpartum

| Characteristic | *p*-value | Test |
| --- | --- | --- |
| Measured rectus diastasis (>30 mm) at 6-weeks postpartum | 0.56 | Pearson’s Chi-square |
| Measured rectus diastasis (>30 mm) at 6-months postpartum | 0.112 | Pearson’s Chi-square |
| Age (years) | 0.126 | Independent *t*-test |
| Height (cm) | 0.455 | Independent *t*-test |
| Weight (kg) | 0.106 | Independent *t*-test |
| BMI (kg/m^2^) | 0.114 | Independent *t*-test |
| Country of birth | 0.056 | Pearson’s Chi-square |
| Ethnicity | 0.01 | Pearson’s Chi-square |
| Language | 0.047 | Pearson’s Chi-square |
| Education level | 0.782 | Pearson’s Chi-square |
| Paid work | 0.017 | Fisher’s exact test |
| Previous diagnosis of depression | 0.207 | Pearson’s Chi-square |
| Previous diagnosis of PTSD | 0.615 | Pearson’s Chi-square |
| Smoking | 0.047 | Pearson’s Chi-square |
| Sleep quality | 0.837 | Pearson’s Chi-square |
| Regular exercise | 0.290 | Pearson’s Chi-square |
| Exercise intensity | 0.455 | Pearson’s Chi-square |
