# Supplementary material for: Prevalence of Symptomatic Established Rectus Diastasis of Parity in Primiparous Women: A Prospective Cohort Study From Early Pregnancy to 1‐Year Postpartum
Source: World J Surg. 2026 Jan 8;50(2):344–52. doi: 10.1002/wjs.70227 (PMC12904848; doi:10.1002/wjs.70227)
Supplement: Supplementary file 4 — Table S3: Summary of delivery characteristics. [file WJS-50-344-s003.docx]

Supplementary Table 3. Summary of delivery characteristics

| Delivery characteristics | Frequency (%) |
| --- | --- |
| Mean (SD; range) gestation at birth (weeks) (*n* = 185) | 39 (1.5) (33 to 42) |
| Mean (SD; range) estimated blood loss (ml) (*n* = 180) | 531.4 (375.2; 100 to 2500) |
| Mean (SD; range) total birthweight (including twins) (g) (*n* = 183) | 3414.3 (544.5; 2150 to 5850) |
| Mean (SD; range) length of stay (*n* = 183) | 2.12 (1.2; 0 to 7) |
| Induction of labour |  |
| Yes | 93 (49.5) |
| No | 95 (50.5) |
| Missing | 1 (0.5) |
| Abnormal cardiotocography |  |
| Yes | 78 (41.3) |
| No | 111 (58.7) |
| Missing | 0 (0) |
| Failure to progress |  |
| Yes | 43 (22.8) |
| No | 146 (77.2) |
| Missing | 0 (0) |
| Foetal distress |  |
| Yes | 40 (21.2) |
| No | 149 (78.8) |
| Missing | 0 (0) |
| Shoulder dystocia |  |
| Yes | 14 (7.4) |
| No | 175 (92.6) |
| Missing | 0 (0) |
| Meconium-stained liquor |  |
| Yes | 21 (11.1) |
| No | 168 (88.9) |
| Missing | 0 (0) |
| Premature delivery |  |
| Yes | 4 (2.1) |
| No | 185 (97.9) |
| Missing | 0 (0) |
| Premature rupture of membranes |  |
| Yes | 24 (12.7) |
| No | 165 (87.3) |
| Missing | 0 (0) |
| Presentation |  |
| Occiput anterior | 138 (76.2) |
| Occiput posterior or transverse | 38 (21) |
| Breech | 4 (2.2) |
| Other | 1 (0.6) |
| Missing | 8 (4.2) |
| Delivery method |  |
| Spontaneous vaginal delivery | 69 (37.7) |
| Elective caesarean section | 12 (6.6) |
| Emergency caesarean section | 55 (30.1) |
| Forceps | 20 (10.9) |
| Vacuum | 27 (14.8) |
| Missing | 6 (3.2) |
| Episiotomy |  |
| Yes | 50 (27.3) |
| No | 133 (72.7) |
| Missing | 6 (3.2) |
| 1st or 2nd perineal tear |  |
| Yes | 46 (24.3) |
| No | 143 (75.7) |
| Missing | 0 (0) |
| 3rd or 4th perineal tear |  |
| Yes | 3 (1.6) |
| No | 186 (98.4) |
| Missing | 0 (0) |
| Labial laceration |  |
| Yes | 6 (3.2) |
| No | 183 (96.8) |
| Missing | 0 (0) |
| Vaginal laceration |  |
| Yes | 1 (0.5) |
| No | 188 (99.5) |
| Missing | 0 (0) |
