# Supplementary material for: Prevalence of Symptomatic Established Rectus Diastasis of Parity in Primiparous Women: A Prospective Cohort Study From Early Pregnancy to 1‐Year Postpartum
Source: World J Surg. 2026 Jan 8;50(2):344–52. doi: 10.1002/wjs.70227 (PMC12904848; doi:10.1002/wjs.70227)
Supplement: Supplementary file 5 — Table S4: Comparison of delivery characteristics of the sample population with state and national rates derived from the National Core Maternity Indicators report [1]. [file WJS-50-344-s008.docx]

Supplementary Table 4. Comparison of delivery characteristics of the sample population with state and national rates derived from the National Core Maternity Indicators report (1)

| Delivery characteristics | Sample | SA | Australia |
| --- | --- | --- | --- |
| Induction of labour | 49.5% | 46.2% | 43.0% |
| Delivery method |  |  |  |
| Spontaneous vaginal delivery | 37.7% | 43.9% | 42.5% |
| Caesarean section | 36.7% | 34.4% | 34.5% |
| Instrumental | 25.7% | 21.6% | 23.0% |
| 3rd or 4th perineal tear after vaginal birth | 2.6% | 4.7% | 4.3% |

References

1. Australian Institute of Health and Welfare. National Core Maternity Indicators. Canberra: AIHW, 2024.
