# Supplementary material for: Prevalence of Symptomatic Established Rectus Diastasis of Parity in Primiparous Women: A Prospective Cohort Study From Early Pregnancy to 1‐Year Postpartum
Source: World J Surg. 2026 Jan 8;50(2):344–52. doi: 10.1002/wjs.70227 (PMC12904848; doi:10.1002/wjs.70227)
Supplement: Supplementary file 6 — Table S5: SF‐36 scores for women with and without symptomatic established rectus diastasis at 12‐month postpartum. [file WJS-50-344-s006.docx]

Supplementary Table 5. SF-36 scores for women with and without Symptomatic Established Rectus Diastasis at 12-months postpartum

|  | Symptomatic Established Rectus Diastasis  (*n* = 26) | | No Symptomatic Established Rectus Diastasis (*n* = 79) | | *t*-test | Effect Size (95% CI) |
| --- | --- | --- | --- | --- | --- | --- |
|  | **Mean** | **SD** | **Mean** | **SD** | ***p*-value** |  |
| Physical Function | 89.8 | 12.4 | 95.6 | 6.9 | 0.016 | 0.67 (0.22 to 1.12) |
| Role Physical | 84.9 | 17.5 | 93.0 | 11.1 | 0.017 | 0.63 (0.17 to 1.08) |
| Bodily Pain | 73.5 | 18.2 | 86.9 | 16.5 | <0.001 | 0.79 (0.33 to 1.25) |
| General Health | 67.7 | 17.6 | 70.9 | 17.9 | 0.213 | 0.18 (-0.26 to 0.62) |
| Vitality | 50.5 | 18.8 | 53.4 | 17.8 | 0.238 | 0.16 (-0.28 to 0.61) |
| Social Function | 85.1 | 16.2 | 90.3 | 14.8 | 0.065 | 0.35 (-0.10 to 0.79) |
| Role Emotional | 89.4 | 16.8 | 89.9 | 17.1 | 0.454 | 0.03 (-0.42 to 0.47) |
| Mental Health | 74.8 | 15.2 | 77.9 | 15.0 | 0.182 | 0.21 (-0.24 to 0.65) |
| Physical Component Score | 50.6 | 6.3 | 54.6 | 4.5 | 0.002 | 0.803 (0.34 to 1.26) |
| Mental Component Score | 46.5 | 9.9 | 46.6 | 9.5 | 0.487 | 0.01 (-0.44 to 0.45) |
