# Supplementary material for: Prevalence of Symptomatic Established Rectus Diastasis of Parity in Primiparous Women: A Prospective Cohort Study From Early Pregnancy to 1‐Year Postpartum
Source: World J Surg. 2026 Jan 8;50(2):344–52. doi: 10.1002/wjs.70227 (PMC12904848; doi:10.1002/wjs.70227)
Supplement: Supplementary file 7 — Table S6: Multiple linear regression model for umbilical inter‐rectus distance at 12‐month postpartum. [file WJS-50-344-s005.docx]

Supplementary Table 6. Multiple linear regression model for umbilical inter-rectus distance at 12-months postpartum

| Variable | Coefficient (B) | Standard error | 95% CI | *p*-value |
| --- | --- | --- | --- | --- |
| Intercept | -51.13 | 23.98 | -98.69 to -3.58 | 0.035 |
| Gestation at birth | 1.95 | 0.61 | 0.73 to 3.16 | 0.002 |
| Failure to progress | 4.58 | 1.90 | 0.82 to 8.35 | 0.018 |
| First- or second-degree perineal tear | -4.69 | 1.87 | -8.39 to -0.99 | 0.014 |
