# Supplementary material for: Prevalence of Symptomatic Established Rectus Diastasis of Parity in Primiparous Women: A Prospective Cohort Study From Early Pregnancy to 1‐Year Postpartum
Source: World J Surg. 2026 Jan 8;50(2):344–52. doi: 10.1002/wjs.70227 (PMC12904848; doi:10.1002/wjs.70227)
Supplement: Supplementary file 8 — Table S7: Binomial logistic regression of risk factors for symptomatic established rectus diastasis at 12‐month postpartum. [file WJS-50-344-s002.docx]

Supplementary Table 7. Binomial logistic regression of risk factors for Symptomatic Established Rectus Diastasis at 12-months postpartum

| Variable | B | Standard Error | *p*-value | Odds Ratio | 95% CI for odds ratio |
| --- | --- | --- | --- | --- | --- |
| BMI | -0.17 | 0.069 | 0.014 | 0.84 | 0.74 to 0.97 |
| Total birthweight (kg) | 1.118 | 0.56 | 0.046 | 3.06 | 1.02 to 9.16 |
| Gestational diabetes | 1.896 | 0.803 | 0.018 | 6.66 | 1.38 to 32.1 |
| Delivery method^¥^ | 0.576 | 0.569 | 0.312 | 1.78 | 0.58 to 5.42 |
| Failure to progress | 0.746 | 0.598 | 0.212 | 2.11 | 0.65 to 6.81 |

^¥ Caesarean birth compared to vaginal birth^
